# Aspirin and its related non-steroidal anti-inflammatory drugs

**DOI:** 10.3402/ljm.v8i0.21569

**Published:** 2013-07-01

**Authors:** Eamon J. Mahdi

**Affiliations:** Department of Haematology, University Hospital of Wales, Cardiff, UK

**Fig. 1:**
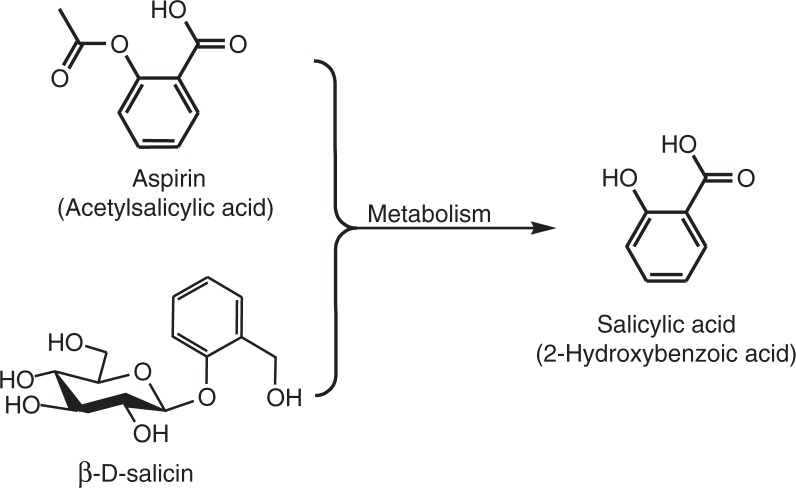
The structures and metabolisms of salicylate compounds.

Aspirin or acetylsalicylic acid has been utilised by physicians for hundreds of years as an analgesic, anti-inflammatory and antipyretic (1). Derived from plant sources, such as the willow tree, it has the ability to induce apoptosis in cancer cells and stimulate angiogenesis (2–4). Earlier research has established that the therapeutic benefit of willow is related to the pro-drug, β-d-salicin, which is metabolised to salicylic acid (SA) in the gastrointestinal system and blood (Fig. 1) (1, 5). Pharmacologically, SA is capable of modulating inflammation via the inhibition of the transcription factor, NF-κB, and subsequently the expression of COX-2 (5). However, aspirin inhibits both COX-1 and -2 irreversibly, thereby inactivating prostanoid cascades for the production of prostaglandins, thromboxanes and prostacyclins, the essential fatty acid signalling molecules (6, 7). Thus, the non-specific mode of action of aspirin suggests the necessity for the development of more specific COX-2 inhibitors. Indeed, our recent research has clearly shown that a number of salicylate-related compounds exhibit modulation of inflammation and are more effective than aspirin (5, 8). For example, 4-hydroxybenzoate zinc was found to specifically inhibit COX-2 *via* the inactivation of the transcription factor NF-κB. Certainly, these were also more effective at inhibiting different cancer cell lines *in vitro* and in primary CLL cells when compared to aspirin (5, 8, 9). Despite a host of pharmacological benefits, aspirin is associated with potential side effects such as peptic ulcers, deafness and dizziness in toxicity, and it is relatively contraindicated in children (10). Therefore, further research into the potential of such aspirin-related compounds is imperative to produce safer and more targeted therapy.

*Eamon J. Mahdi* Department of Haematology University Hospital of Wales, Cardiff, UK
